# Bibliometric and visualized analysis of 2014–2024 publications on therapy for diabetic peripheral neuropathy

**DOI:** 10.3389/fnins.2024.1434756

**Published:** 2024-11-06

**Authors:** Baitian Fu, Ning Luo, Yichen Zeng, Yutian Chen, Low Je Wie, Jianqiao Fang

**Affiliations:** ^1^The Third Clinical Medical School, Zhejiang Chinese Medicine University, Hangzhou, China; ^2^Institute of International Education of Zhejiang Chinese Medical University, Hangzhou, China

**Keywords:** diabetic peripheral neuropathy, therapy, treatment, bibliometric analysis, CiteSpace

## Abstract

**Background:**

This research aimed to examine the global developing patterns in the treatment of diabetic peripheral neuropathy (DPN) using a bibliometric analysis of published literature.

**Methods:**

We extracted publication data from papers published between 2014 and 2024 using a specific topic search in the “Web of Science Core Collection” (WoSCC) database. Various metrics, such as the number of papers, citations, authors, countries, institutions, and references, were collected for analysis. To further explore the data, CiteSpace was employed to examine co-citation patterns among authors, identify collaborative efforts between countries and institutions, and uncover emerging trends using burst keywords and reference analysis.

**Results:**

The study encompassed 2,488 publications that exhibited an increasing trend in annual output. Notably, the journal *PAIN*, the United States, the Pfizer institution, and the author Feldman, EvaL emerged as the most prolific contributors to this research domain. The term “placebo-controlled trial” was the most prominent burst keyword from 2014 to 2017, whereas “spinal cord stimulation” held this distinction in the recent 5-year span. Furthermore, the publication titled “Pharmacotherapy for neuropathic pain in adults: a systematic review and meta-analysis-2015” demonstrated the highest burst in terms of references.

**Conclusion:**

This study is the first to objectively reveal the current hotspots and trends in DPN treatment. The results indicate that drug therapy remains the primary first-line treatment for DPN and that future research on DPN treatment will likely focus on “spinal cord stimulation” and “pain management.” These findings provide valuable insights into DPN treatment.

## 1 Introduction

Diabetic peripheral neuropathy (DPN) is a prevalent chronic complication in patients with type 1 and type 2 diabetes (Pop-Busui et al., 2017) that causes damage to peripheral nerves caused by prolonged high blood sugar levels. It primarily manifests as numbness, pain, tingling, burning sensations, or loss of sensation in the distal extremities. Common symptoms include numbness, pain in the extremities, formication, a warp-like sensation in the legs, and diminished tendon reflexes (Elafros et al., 2022). Research indicates that over half of diabetic patients may potentially experience DPN (Stino and Smith, 2017). Sensory disorders in DPN patients are primarily attributed to distal axonal degeneration and atrophy of sensory neurons within the dorsal root ganglion (DRG), which affects the proximal extremities and central chest (Miyashita et al., 2023; Kobayashi and Zochodne, 2018).

As global research on DPN has intensified over the past decade, from 2014 to 2024, various guidelines and studies have proposed relevant treatment methods for DPN. Drug therapy currently stands as the primary treatment for DPN, according to international guidelines (Callaghan et al., 2012) from organizations such as the American Academy of Neurology and the European Federation of Neurological Societies. These guidelines recommend calcium channel α2-d ligands (e.g., gabapentin and pregabalin) and selective serotonin–norepinephrine reuptake inhibitors (SNRIs) as first-line medications for DPN (Yang et al., 2022). Additional drugs include opioid analgesics, capsaicin, and lidocaine. However, drug treatment may present challenges, such as high recurrence rates, drug dependence, resistance, and adverse reactions such as ataxia, vertigo, blurred vision, lethargy, constipation, fatigue, and nausea (Ametov et al., 2003). While surgical procedures are an option, they pose the risk of significant trauma, neurovascular injury, and other adverse events (Jang and Oh, 2023). Therefore, there is an urgent need for a systematic review and comprehensive summary of DPN treatment approaches.

Bibliometrics is a widely used method in medical research for effectively examining and illustrating research trends (Chen, 2006; Liu et al., 2021). It enables researchers to gain a comprehensive understanding of the current status and evolving patterns within the field. In addition, it helps in the identification of potential research areas and significant issues, the assessment of research quality and impact, the understanding of research trajectories and methodologies, and the fostering of academic interactions and collaborations, all of which contribute to the advancement of the field. Moreover, bibliometric analysis has been applied in various research domains, such as artificial intelligence (AI), industry, and medicine (Guo et al., 2020; Colares et al., 2020). Past studies have illustrated bibliometric analyses on topics such as Qigong, including its relation to diabetes (Zhang et al., 2020). However, there is a lack of studies that focus on the bibliometric analysis of trends in therapy for DPN.

Consequently, this study used bibliometric analysis to identify crucial evidence in the treatment of DPN, aiding researchers in understanding the scholarly foundations and current research trajectory within this domain.

## 2 Materials and methods

### 2.1 Data sources and search strategy

The Web of Science Core Collection (WoSCC) is a comprehensive academic information resource spanning multiple disciplines (Lv et al., 2023). In January 2024, a total of 4,741 published papers were retrieved from it under the topics of “diabetic peripheral neuropathy and therapy,” “diabetic peripheral neuropathy and treatment,” or “diabetic peripheral neuropathy and remedy” in the Science Citation Index Expanded (SCI-EXPANDED) and the Social Sciences Citation Index (SSCI). The search strategy used was: TS = [(diabetic peripheral neuropathy) AND (therapy)] OR TS = [(diabetic peripheral neuropathy) AND (treatment)] OR TS = [(diabetic peripheral neuropathy) AND (remedy)]. The publication date range was limited to 2014–2024 to reflect the most recent developments in this research area.

### 2.2 Data analysis

**Figure 1:**
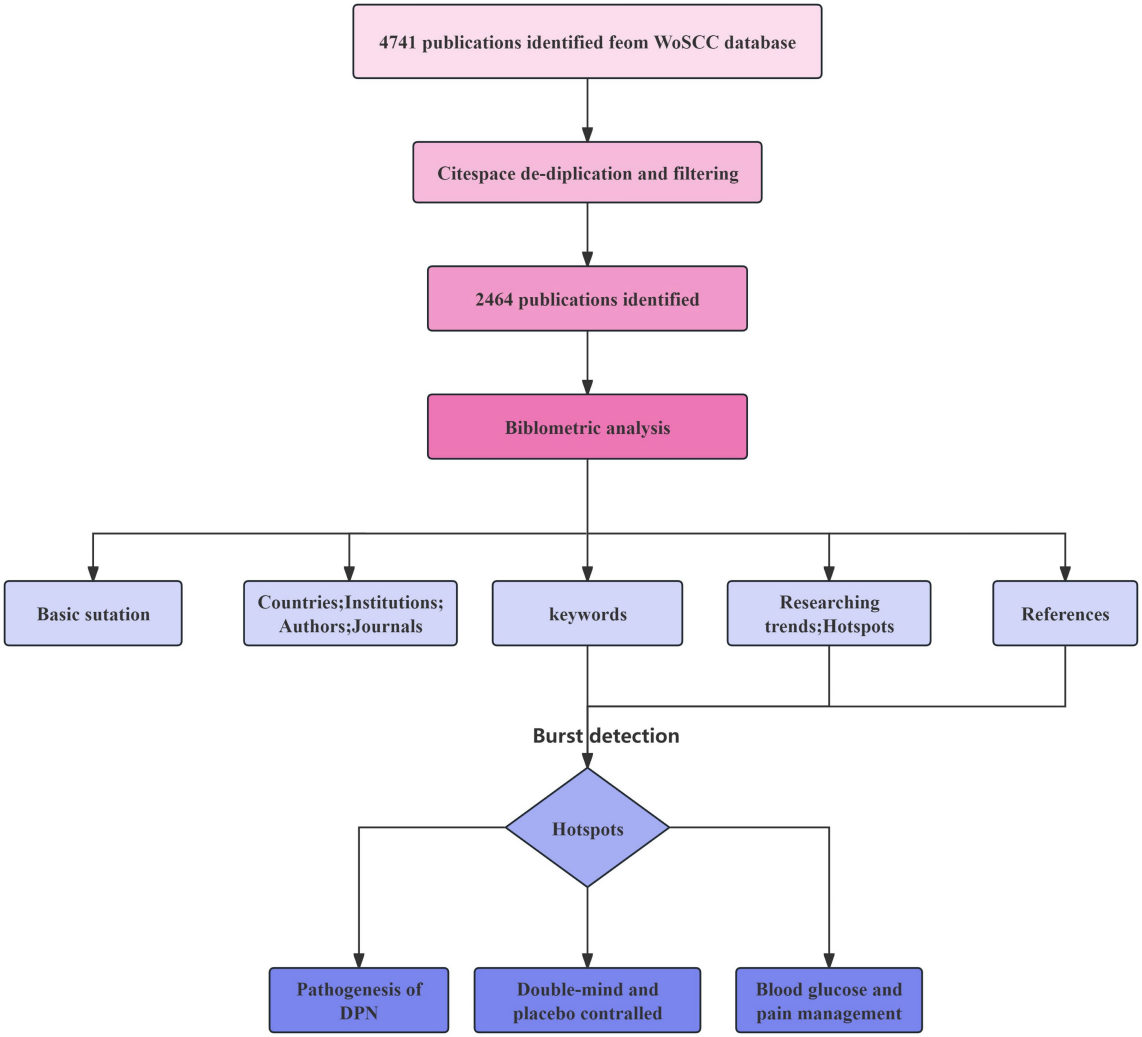
Flowchart of the review.

The author retrieved documents from the Web of Science Core Collection (WoSCC) using the specified retrieval mode. The data were exported to a plain text file, including full records and cited references, and saved in download-txt format. Disagreements were resolved through a consensus. The valid data were then tranferred to Microsoft Excel 2020, CiteSpace (6.2. R6), and GraphPad Prism 9.0. A review flowchart is provided in Figure 1, and the complete records and references of all included publications were downloaded for further analysis.

The WoSCC website provides essential features for search results, including the count of papers, citations, authors, countries, references, and more. Consequently, the WoSCC was used for the initial examination of publication trends in physical activity therapy for diabetes.

CiteSpace is a valuable visual analytic tool for conducting bibliometric analysis (Chen, 2006; Chen, 2018). By utilizing bibliographic data from the WoSCC, it enables the detection of bursts, intermediate centrality, and heterogeneous networks, as well as the identification and tracking of critical paths and milestone studies in professional development (Lv et al., 2023). Moreover, CiteSpace helps identify emerging trends and transient patterns at the forefront of research, thereby enhancing the reproducibility, efficiency, and exploitability of bibliometric analysis by highlighting the most representative published papers (Chen and Song, 2019). In this study, CiteSpace (6.2.R6) was used to analyze institutions, countries, keywords, and references within clusters, creating network maps where the size of the circle represents contribution and the number of lines represents relationship strength. Furthermore, the tool was used to analyze citation bursts of keywords and references, providing insights into emerging trends. Descriptive statistics, including ranks, frequency, attributes, and centrality, were conducted using Microsoft Word 2019, while GraphPad Prism 9.0 was used for analyzing and visualizing annual publication outputs.

CiteSpace has several key advantages over other bibliometric software, including effective visualization of knowledge structures, unique burst detection for identifying emerging trends, and robust clustering and timeline analysis. Its strength lies in mapping interdisciplinary knowledge flow while supporting multiple data sources, which enables flexible citation analysis. In addition, its user-friendly interface makes it a powerful tool for researchers to analyze and visualize complex academic networks with ease.

## 3 Results

### 3.1 Annual literature volume and growth

**Figure 2:**
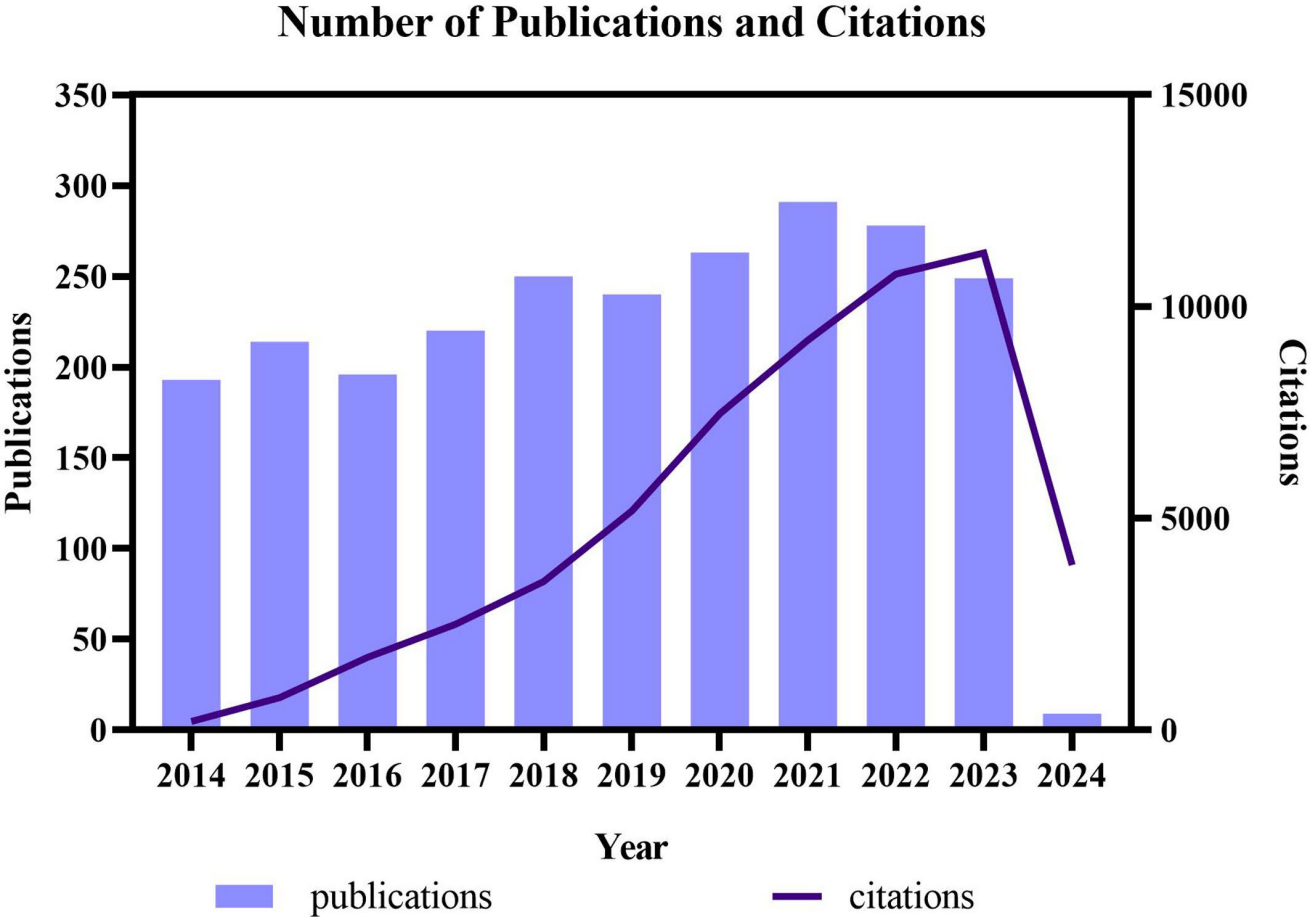
Overview of research publications and citation counts in the treatment of diabetic peripheral neuropathy research from 2014 to 2023.

This analysis included 2,488 publications based on primary research in the WoSCC database. Over the last decade, the annual publishing distribution exhibited a generally consistent upward trend with fluctuations. This trend can be categorized into three segments: between 2014 and 2017, the average publication count was 205.75; from 2018 to 2021, there was an increase in publications, with an average count of 261; and from 2021 to 2023, the publication trend remained relatively stable. In addition, the citation frequency of these publications increased significantly from 2014 to 2024 (Figure 2).

### 3.2 Country and institute analysis

**Figure 3:**
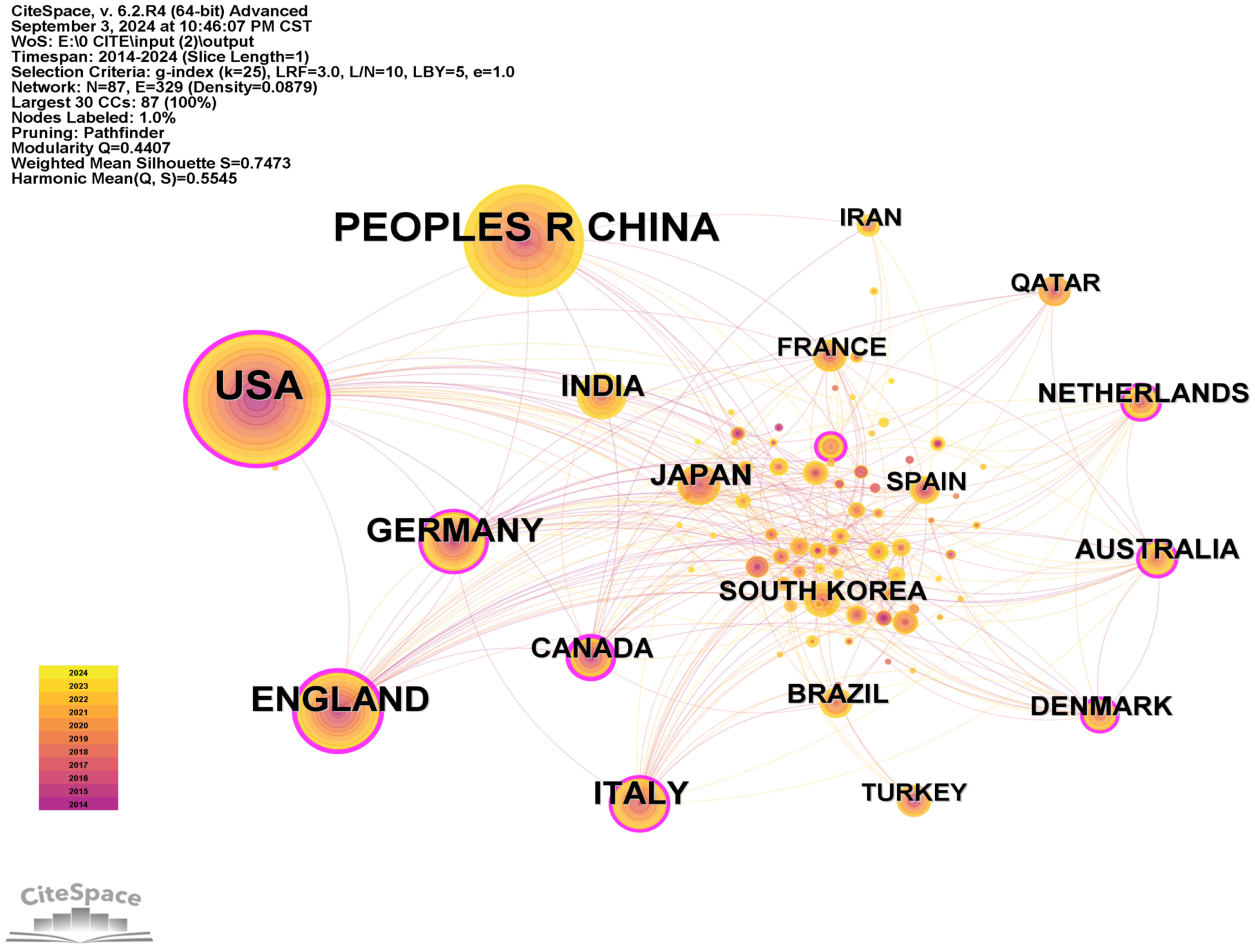
Visualization map of the countries that contributed to research on therapy for diabetic peripheral neuropathy. Each colored solid circle represents a country/region, while the lines between them represent collaborative relationships.

**Figure 4:**
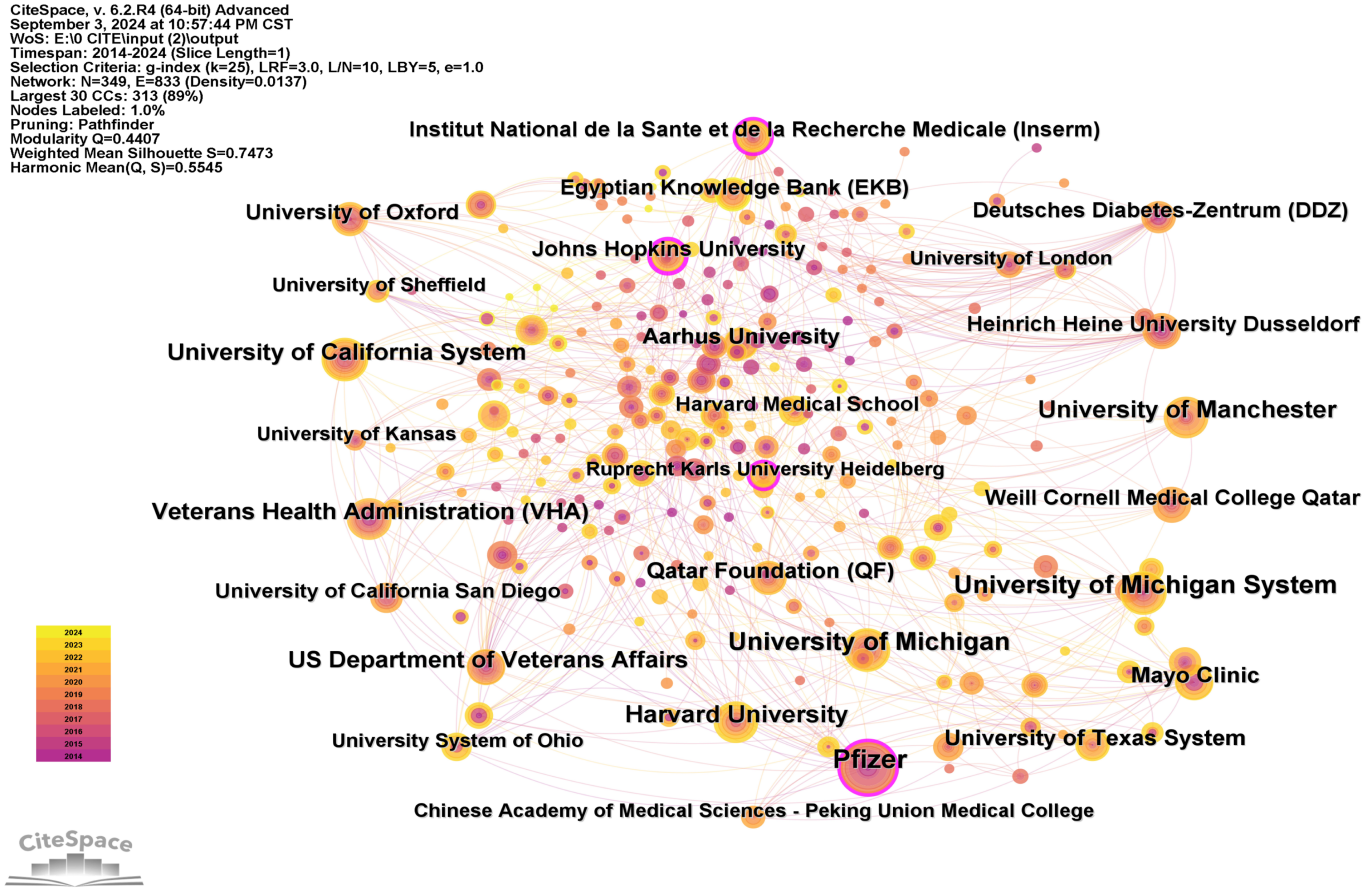
Visualization map of institutions contributed to therapy for diabetic peripheral neuropathy research. Each colored solid circle represents an institution, and the lines between them represent collaborative relationships.

The WoSCC provided important information on the included publications, such as countries and institutions. Table 1 shows the leading 10 countries focused on therapy for DPN. The United States predominated with 713 publications (28.92%), the highest total citations (22,658), and an H-index of 73. Canada had the highest average citations per paper (50.79), while the United States, England, and Canada were ranked highest in centrality, with scores of 0.28, 0.24, and 0.23, respectively. Centrality is a metric that reflects the importance of nodes in a network. Using CiteSpace, a network map illustrating the countries’ contributions was generated, with the size of the circles corresponding to their impact. The visual representation in Figure 3 highlights the United States, China, and England as the primary contributors. A subsequent analysis, as shown in Table 2, identified the top 10 institutions engaged in DPN treatment research, with Pfizer leading with 59 publications (2.62%), followed by the University of Michigan with 53 publications (2.35%), and the University of Manchester with 45 publications (1.99%). The network mapping of the institutions positioned Pfizer, the University of Michigan, and the University of Manchester as the most influential entities, as shown in Figure 4. From 2021 to 2024, significant citation bursts were observed for Harvard University, the University of Texas System, Mayo Clinic, and Harvard Medicine School, suggesting that these institutions heightened research contribution from these in the near future.

**Table 1:** Top 10 prolific countries in therapy for diabetic peripheral neuropathy research.

Rank	Country	Count	Percentage (%)	Centrality	H-index	Citations per paper	Citations WoS
1	USA	713	28.92	0.28	73	31.78	22,658
2	The People’s Republic of China	559	22.68	0.04	42	13.94	7,792
3	England	235	9.53	0.24	48	44.17	10,381
4	Germany	169	6.86	0.12	38	43.4	7,334
5	Italy	134	5.44	0.17	31	30.1	4,034
6	Japan	99	4.02	0.02	23	17.53	1735
7	Canada	82	3.33	0.23	31	50.79	4,165
8	India	122	4.95	0.06	24	23.47	2,863
9	The Netherlands	70	2.84	0.11	23	27.89	1952
10	Australia	78	3.16	0.12	23	25.13	1960

**Table 2:** Top 10 prolific institutions in therapy for diabetic peripheral neuropathy research.

Rank	Institution	Count	Percentage (%)	Centrality
1	Pfizer	59	2.62	0.05
2	University of Michigan	53	2.35	0.03
3	University of Manchester	45	1.99	0.08
4	Harvard University	43	1.91	0.15
5	US Department of Veterans Affairs	40	1.77	0.03
6	Veterans Health Administration (VHA)	37	1.64	0.03
7	University of California System	36	1.60	0.08
8	Qatar Foundation (QF)	33	1.46	0.01
9	Aarhus University	32	1.42	0.05
10	University of Texas System	31	1.37	0.12

### 3.3 Analysis of the published journals

The research in this study referenced papers published across diverse fields such as molecular biology, genetics, medicine, nursing, rehabilitation, and health, comprising 10 different types of literature, as shown in Table 3. Notably, the majority of these publications (69.89%) were research articles, followed by review articles (27.92%). The WoSCC provided important information regarding the journals in which these papers were published. Table 4 shows the top 10 journals based on publication and citation counts, with impact factors ranging from 1.9 to 10.9. The impact factor is a metric that indicates the average annual citations received by a journal’s recent articles and is commonly used to evaluate a journal’s influence within its field. Our analysis indicated that the journals with higher publication rates were more inclined to accept research articles on inflammation in DPN therapy compared to those with lower publication rates. Moreover, our study found that the top 10 prestigious journals, with an impact factor of at least 3.0, accounted for 9.4% of the total literature output in the field over the past decade.

**Table 3:** Types of journal articles (the original results were collected by the WoS analysis tool).

Document types	Count	Percentage (%)
Article	1722	69.89
Review	688	27.92
Other	54	2.19

**Table 4:** Top 10 journals by publication (left) and top 10 cited journals (right).

Journal	Number of publications	Total times cited	IF (5 years)	Journal	Number of publications	Total times cited	IF (5 years)
Pain	35	1778	7.7	Cochrane Database of Systematic Reviews	18	1931	10.9
Journal of Pain Research	34	561	3.2	Pain	35	1778	7.7
Frontiers in Endocrinology	31	431	5.9	Diabetes Care	24	1,641	16
Medicine	31	168	1.9	PLoS One	29	1,007	3.8
Journal of Diabetes Research	29	708	4.7	Journal of Diabetes Research	29	708	4.7
PLoS One	29	1,007	3.8	Pain Medicine	26	659	3.4
Diabetes Research and Clinical Practice	26	471	6.1	Diabetes/Metabolism Research and Reviews	24	608	6.3
International Journal of Molecular Sciences	26	381	6.2	Diabetes	23	554	8.2
Journal of Diabetes and its Complications	26	449	3.1	Pain Practice	21	536	2.9
Pain Medicine	26	659	3.4	Journal of Diabetes Investigation	19	482	3.7

The Cochrane Database of Systematic Reviews emerged as the most frequently cited journal with 1,931 citations, leading the ranking ahead of *Pain* (1,778 citations), *Diabetes Care* (1,641 citations), and *PLoS One* (1,007 citations). *The Cochrane Database of Systematic Reviews* holds a central position within this field (IF, 5 years = 10.9, 1778 citations).

### 3.4 Author analysis

**Figure 5:**
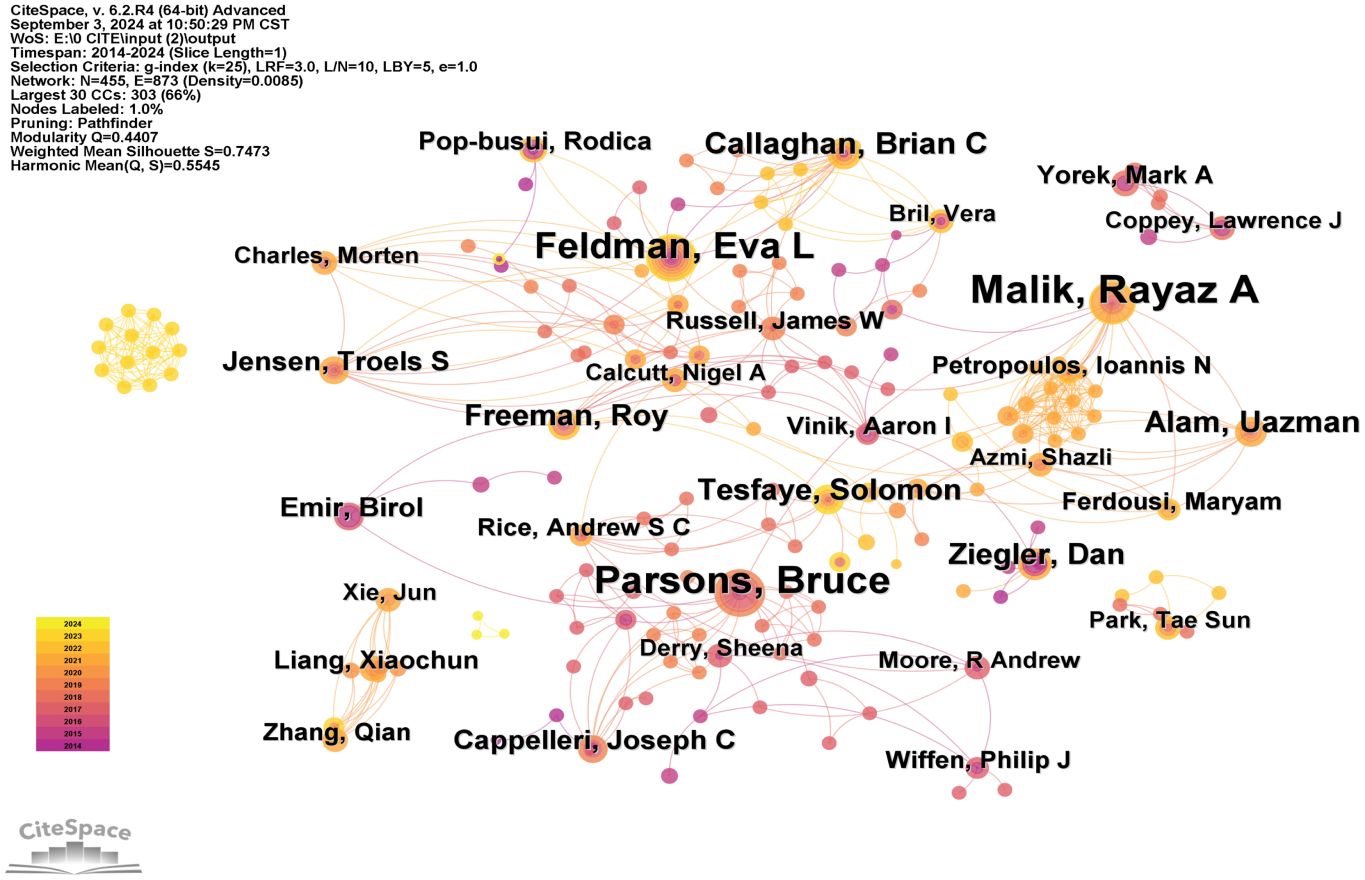
Network map of Citation -authors contributed to therapy for diabetic peripheral neuropathy research. Each colored solid circle represents a author, and the lines between them represent collaborative relationships.

The WoSCC provides key information about the authors of academic publications. Table 5 shows the top 10 authors who have been recognized as accomplished researchers and integral team members. Feldman, Eva L. led the list with 38 publications (6.02%), followed by Parsons Bruce with 36 publications (5.70%), Ziegler, Dan with 30 publications (4.75%), Malik, Rayaz A. with 29 publications (4.60%), and Tesfaye, Solomon with 27 publications (4.27%). Notably, Parsons, Bruce has the highest centrality score of 0.06, followed by Malik, Rayaz A, with a centrality score of 0.05. The citation–author network, shown in Figure 5, highlighted the authors who made significant contributions, with a focus on the increasing impact of Tesfaye Solomon’s study from 2021 to 2024.

**Table 5:** Top 10 prolific authors related to the treatment of diabetic peripheral neuropathy.

Rank	Author	Year	Percentage (%)	Count	Citations WoS	Centrality
1	Feldman, Eva L	2014	6.02	38	2,893	0.02
2	Parsons, Bruce	2015	5.70	36	594	0.06
3	Ziegler, Dan	2014	4.75	30	1,148	0.04
4	Malik, Rayaz A	2014	4.60	29	1,035	0.05
5	Tesfaye, Solomon	2014	4.27	27	864	0.04
6	Alam, Uazman	2014	3.49	22	927	0.02
7	Yorek, Mark	2015	3.32	21	544	0.00
8	Callaghan, Brian C	2015	2.85	18	1,650	0.02
9	Pop-Busui, Rodica	2014	2.69	17	1795	0.00
10	Jensen, Troels Staehelin	2017	2.53	16	1927	0.02

### 3.5 Keyword co-occurrence and clustering analysis

**Figure 6:**
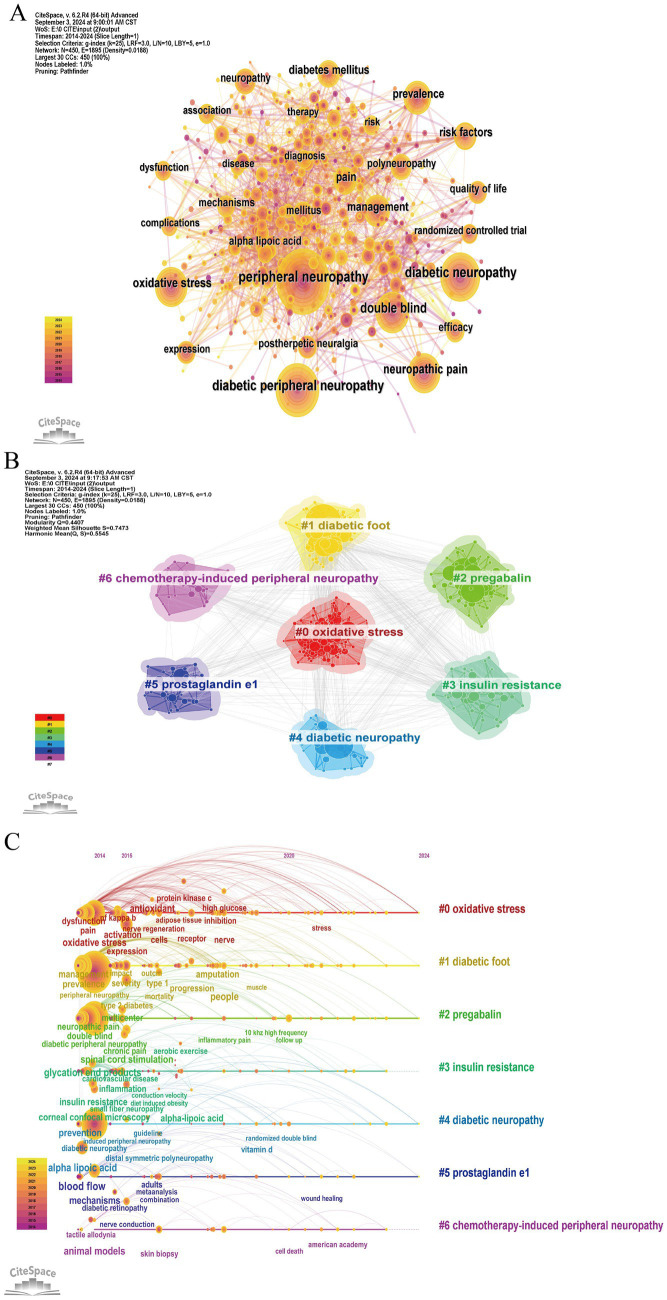
**(A)** Network map of keywords contributed to therapy for diabetic peripheral neuropathy research, each colored solid circle represents a keyword, and the lines between them represent collaborative relationships; **(B)** Cluster analysis of keyword co-appearance; **(C)** The timeline view of keywords co-appearance.

The network visualization produced by CiteSpace displayed prominent topics such as “peripheral neuropathy, diabetic peripheral neuropathy, diabetic neuropathy, oxidative stress, neuropathy pain, double mind, prevalence, management,” with larger circles (Figure 6A) indicating their high frequency and significance in the realm of “therapy for diabetic peripheral neuropathy,” Through the application of the LLR algorithm, seven keyword clusters were identified, with a Modular Q value (Q value) = 0.441 > 0.3 and a mean silhouette value (S value) = 0.7568 > 0.7, affirming the robustness of the clustering pattern. Notably, oxidative stress, diabetic foot, and pregabalin have emerged as pivotal topics in the research domain of DPN (Figure 6B). Figure 6C shows a timeline viewer based on CiteSpace software that shows the keyword cluster related to research on the therapy of DPN from 2014 to 2024. It shows the timeline distribution of the keyword cluster analysis. Nodes on the same line represent the same cluster. The closer a node is to the right, the more frequently it appears in recent research. There are seven clusters in the diagram: Cluster #0 (oxidative stress), Cluster #1 (diabetic foot), Cluster #2 (pregabalin), Cluster #3 (insulin resistance), Cluster #4 (diabetic neuropathy), Cluster #5 (prostaglandin e1), and Cluster #6 (chemotherapy-induced peripheral neuropathy).

### 3.6 Keyword burst citation

**Figure 7:**
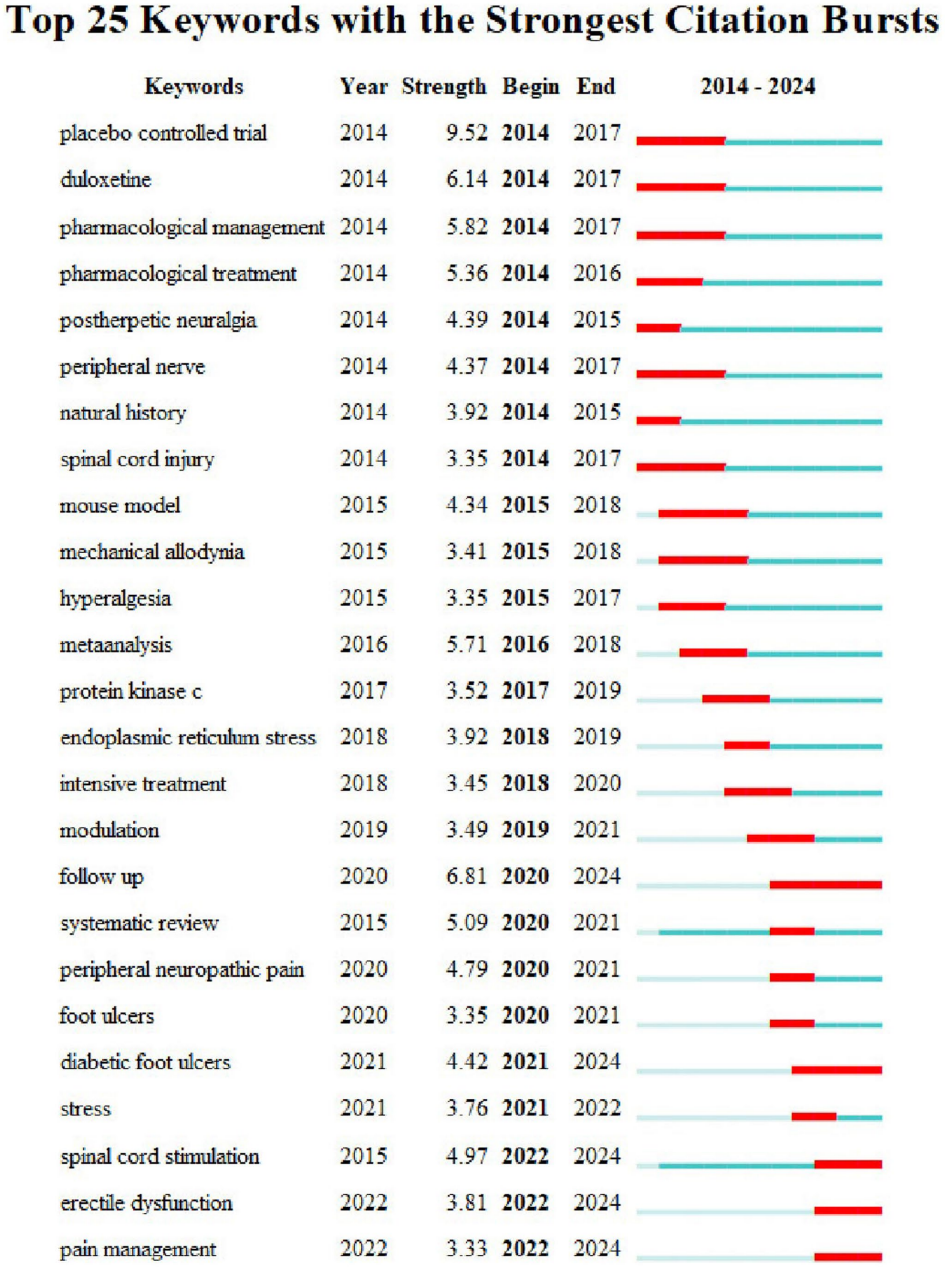
Top 25 keywords with the strongest citation bursts in the treatment of diabetic peripheral neuropathy research.

In addition, Figure 7 illustrates a surge in the citations of the top 25 keywords over the past decade. Keywords such as “placebo-controlled trial,” “duloxetine,” “pharmacological management,” “pharmacological treatment,” and “postherpetic neuralgia” have been extensively researched since 2014 or earlier. Keyword burst strength in CiteSpace measures the extent to which a keyword experiences a sudden increase in attention or citation frequency over a specific time period. Notably, the term “placebo-controlled trial” has the highest strength of 9.52. However, between 2021 and 2022, new keywords with strong citations emerged, including “diabetic foot ulcers,” “stress,” “spinal cord stimulation,” “erectile dysfunction,” and “pain management.” This suggests a shift in hot topics in this field of research, potentially indicating future popular keywords.

Numerous studies have established the correlation between placebo-controlled trials, duloxetine, pharmacological management, pharmacological treatment, and postherpetic neuralgia in relation to DPN treatment, emphasizing their significant role in managing DPN. Researchers are exploring various approaches to enhance the prognosis of DPN patients. Advancements in molecular biology technology are expected to introduce innovative therapeutic methods in clinical settings, hence expanding treatment options for DPN.

### 3.7 Co-citation clustering analysis of the references and burst citation

**Figure 8:**
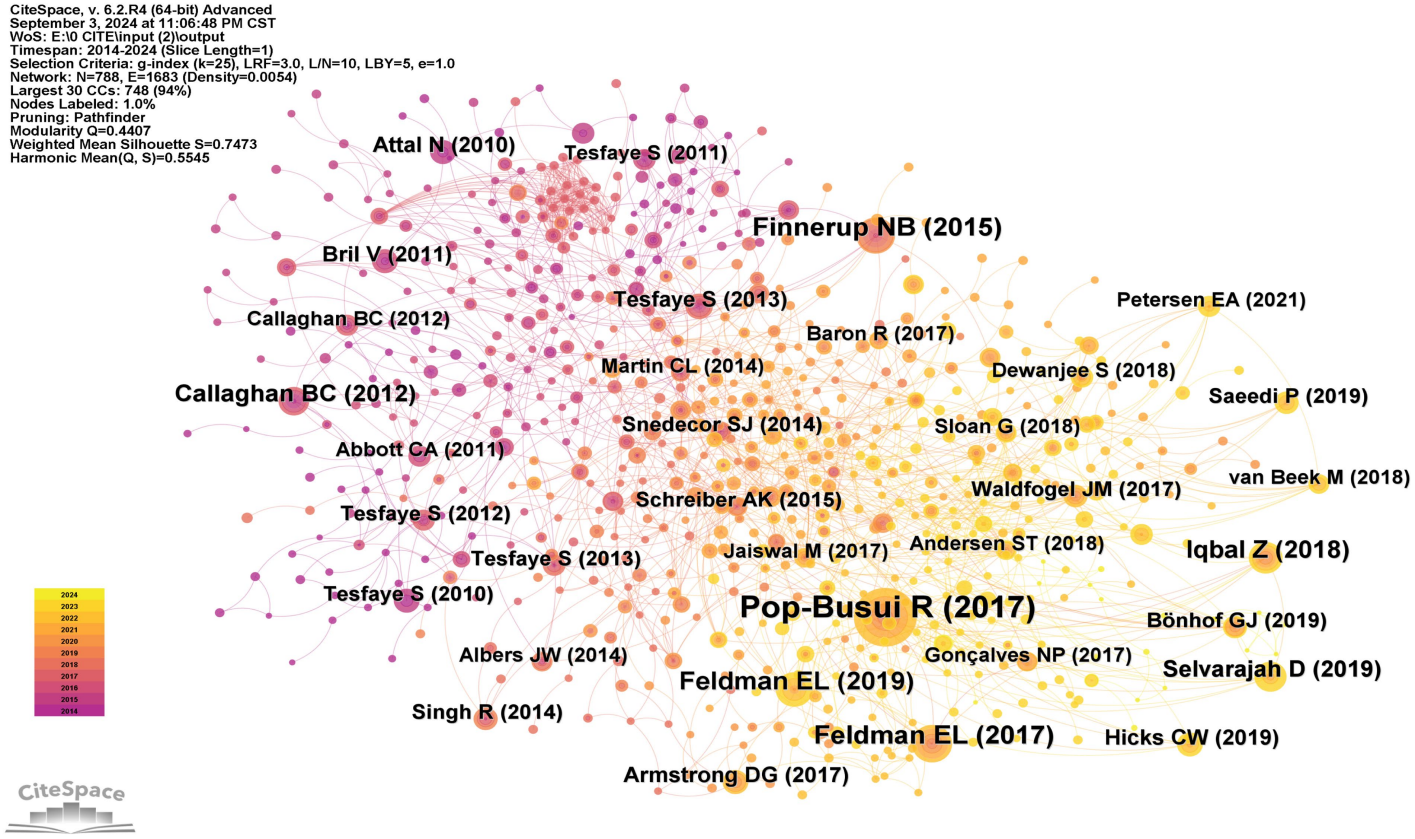
Network map of references contributed to therapy for diabetic peripheral neuropathy research. Each colored solid circle represents a reference, and the lines between them represent collaborative relationships.

**Figure 9:**
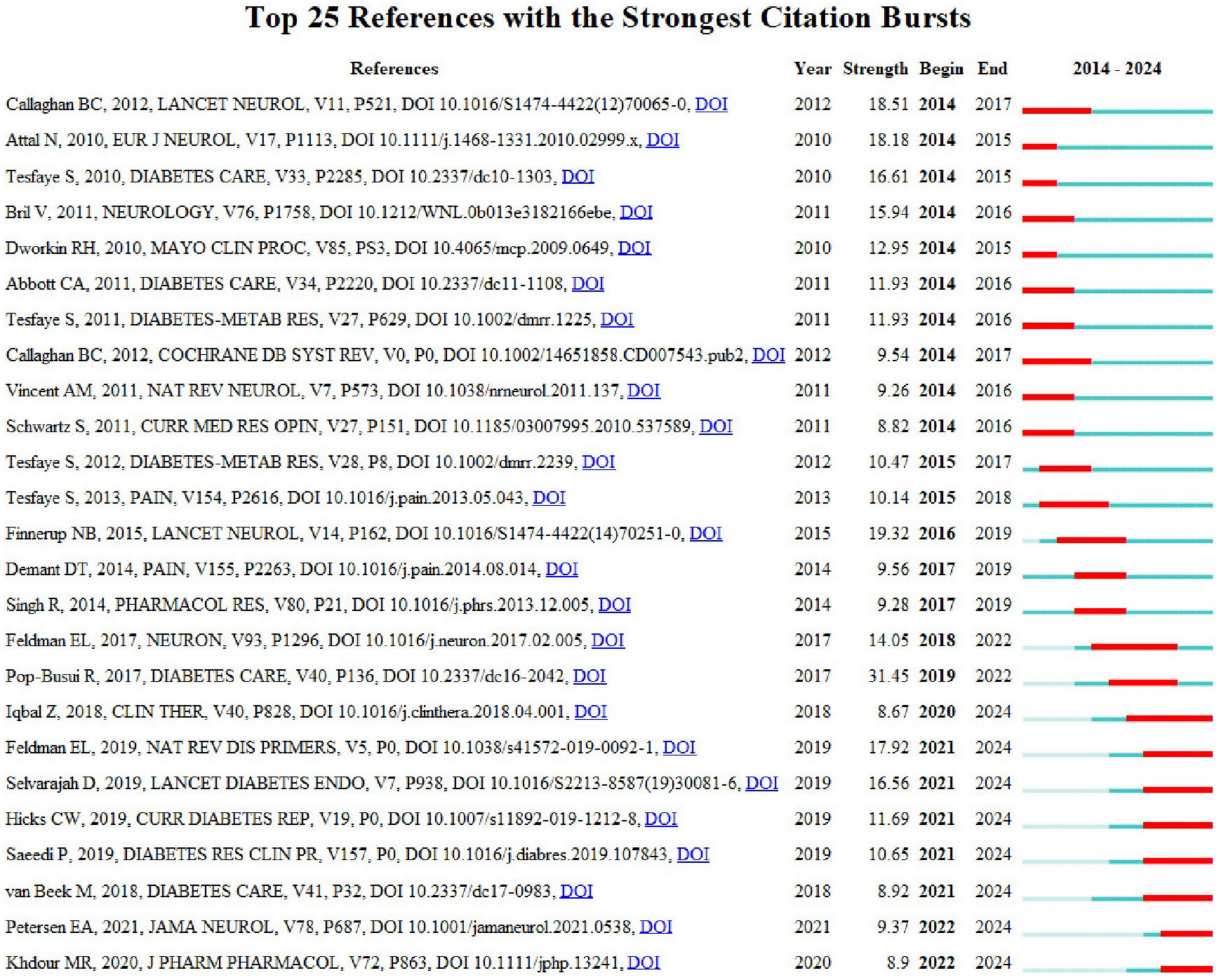
Top 25 references with the strongest citation bursts in the treatment of diabetic peripheral neuropathy.

The network map of co-citation sites generated by CiteSpace depicted 760 nodes and 3,123 links, as illustrated in Figure 8. Table 6 presents the top five cited papers, with the most frequently cited paper being “Diabetic Neuropathy: A Position Statement by the American Diabetes Association” (Pop-Busui et al., 2017), which was cited 185 times and plays a significant role in guiding the diagnosis and treatment of diabetic peripheral neuropathy. The aforementioned paper was closely followed by “Pharmacotherapy for neuropathic pain in adults: a systematic review and meta-analysis” (Finnerup et al., 2015), which strongly recommends certain medications as first-line treatments for neuropathic pain. In addition, “New Horizons in Diabetic Neuropathy: Mechanisms, Bioenergetics, and Pain” (Feldman et al., 2017) reviews the structural components of the peripheral nervous system, the pathways contributing to peripheral nerve injury in diabetes, and recent advances in biotechnology and bioinformatics. It highlights the rapid expansion of our knowledge regarding the mechanisms contributing to neuropathic pain in diabetes. These developments in understanding the pathogenesis of diabetic neuropathy are crucial for advancing mechanism-based therapies. Figure 9 provides the analysis of the top 25 burst-cited literature, which provides insights into potential future research directions.

**Table 6:** Top five prolific cited references in therapy for diabetic peripheral neuropathy research.

Rank	Cited references	Count	Year	Centrality
1	Diabetic neuropathy: a position statement by the American diabetes association	185	2017	0.00
2	Pharmacotherapy for neuropathic pain in adults: a systematic review and meta-analysis	91	2015	0.00
3	New Horizons in diabetic neuropathy: mechanisms, bioenergetics, and pain	81	2017	0.00
4	Diabetic neuropathy	74	2019	0.00
5	Diabetic peripheral neuropathy: epidemiology, diagnosis, and pharmacotherapy	60	2018	0.00

## 4 Discussion

### 4.1 General information

The research examined 2,488 publications on therapy for DPN from the WoSCC that were published between 2014 and 2024. These publications were analyzed using CiteSpace to assess their distribution across countries, institutions, journals, and authors. The analysis revealed a steady year-by-year increase in the number of publications, indicating a growing interest in research on DPN therapy.

In terms of global contributions to DPN treatment research, the United States and China have published the most publications. However, both average and total citation counts are higher in the United Kingdom and the United States than in China. This indicates that, despite China’s increasing publication output, the quality and impact of its research still lag behind those of the U.S. and the U.K. Therefore, China should focus on improving the quality and influence of its publications in this field. In addition, the U.S. and the U.K. lead international collaborations in research on DPN treatment.

The visualization analysis of the publishing institutions revealed that Pfizer and the University of Michigan in the U.S. are leading contributors to DPN treatment research. Notably, most of the top 10 institutions are based in the U.S., underscoring the country’s dominance in this field. However, this concentration of research efforts may limit the exchange of complementary strengths, thereby slowing the overall development of the field. To address this, other countries should actively seek out specialist talent or send researchers from national or institutional levels to leading institutions, such as Pfizer or the University of Michigan, for targeted training and collaboration.

By analyzing the authors, key experts in the study of DPN treatment can be identified. As shown in Table 5 and Figure 5, Feldman et al. is a key expert in this field. Her highly cited papers primarily discuss the concepts and pathogenesis of DPN, as well as current treatment methods, and propose inflammation as a target for DPN treatment (Feldman et al., 2017; Feldman et al., 2019; Pop-Busui et al., 2016). Analyzing these papers provides insights into the current research directions for DPN treatment.

Centrality reflects the connections between authors, and Parsons, Bruce (0.06) and Malik, Rayaz A (0.05) had the highest centrality scores. This indicates that both authors have close collaborations with other researchers and are heavily involved in DPN treatment-related research. Co-citation frequency reflects, to some extent, the intrinsic scientific value and research background of the literature, as well as the influence of the authors in the field. Translating the papers of highly co-cited authors can help provide a comprehensive understanding of the specific area.

Furthermore, Feldman, Eva L. (2,893) and Jensen, Troels Staehelin (1,927) had the highest citation frequencies, indicating that their views and research findings are widely recognized and valued by the academic community. Their contributions have had a significant impact on the field. Notably, Jensen, Troels Staehelin proposed principles for the diagnosis and treatment of neuropathic pain (Gilron et al., 2015), which provide valuable guidance for the treatment of DPN.

In the analysis of the top 10 journals with the highest number of publications, “*PAIN*” ranked first globally, significantly outperforming other journals in terms of IF, publication volume, and citation frequency. Therefore, in the field of DPN treatment, scholars can conveniently share their achievements and perspectives in this journal, fostering discussion and intellectual exchange with peers to enhance their academic proficiency and research capabilities. Figure 9 highlights the journals experiencing citation bursts, with “*Diabetes Care*,” “*JAMA Neurology*,” and “*Journal of Pharmacy and Pharmacology*” emerging as recent trending publications. This suggests that articles published in these journals are likely to address current hot topics related to DPN treatment. Highly cited papers provide substantial information and influence, laying the theoretical and knowledge foundations for future research.

The three most cited articles were “Diabetic Neuropathy: A Position Statement by the American Diabetes Association” (Pop-Busui et al., 2017), “Pharmacotherapy for Neuropathic Pain in Adults: A Systematic Review and Meta-Analysis” (Finnerup et al., 2015), and “New Horizons in Diabetic Neuropathy: Mechanisms, Bioenergetics, and Pain” (Feldman et al., 2017). Collectively, these articles emphasize that the most effective current approach to DPN treatment is glycemic control and the management of neuropathic symptoms. Therefore, future research on DPN treatment should continue to focus on these two areas.

### 4.2 Hotspots and frontiers

A keyword co-occurrence network and its overlay map indicate research hotspots and frontiers in the field of DPN therapy. In addition, a keyword tree diagram can significantly indicate the weight of clusters and keywords, while a timeline viewer of keywords can reflect the evolution of research hotspots over time. The analysis of the keywords network map indicated that “peripheral neuropathy, diabetic peripheral neuropathy, diabetic neuropathy, oxidative stress, neuropathy pain, double mind, prevalence, management” were prominently featured with large circles (Figure 6A). This underscores the predominant focus in research on therapy for DPN, particularly concerning pain-related treatments, blood glucose management, and pathogenesis, especially oxidative stress. Furthermore, Figure 7 illustrates a surge in citations for the top 25 keywords over the past decade. Notably, keywords such as “placebo-controlled trial, duloxetine, pharmacological management, pharmacological treatment, and postherpetic neuralgia” have been extensively researched since at least 2014. Conversely, from 2021 to 2022, new keywords emerged with strong citation counts, including “diabetic foot ulcers, stress, spinal cord stimulation, erectile dysfunction, and pain management.” This evolution suggests a shift in research focus within the field, potentially indicating future trending keywords.

Based on the results of the keyword co-occurrence, clustering, and timeline analysis, we can explore current and future treatment methods for DPN in detail from the following three aspects. First, oxidative stress is one of the mechanisms behind the onset of DPN. In hyperglycemic conditions, the polyol metabolic pathway is activated, leading to increased aldose reductase activity (Lv et al., 2024). This triggers an acceleration in the glycation process, resulting in a significant amount of advanced glycation end products (AGEs) (Volpe et al., 2018). Upon binding with their receptors, reactive oxygen species are released, resulting in oxidative stress. Simultaneously, the activation of the nuclear factor-κB transcription factor triggers inflammation, contributing to segmental demyelination of peripheral neurons (Lin et al., 2023). In addition to the direct damage caused by high blood sugar levels to peripheral nerve axons, impaired blood–nerve barrier and Schwann cells disrupt the homeostasis of peripheral nerves (Takeshita et al., 2020). Recent studies have suggested that a systemic deficiency in serine can disturb lipid balance, potentially leading to dyslipidemia, which emerges as a novel risk factor for peripheral neuropathy (Handzlik et al., 2023). Inflammatory factors such as heat shock proteins (HSPs), polyADP ribose polymerase, tumor necrosis factor *α* (TNF-α), and immune cells play a vital role in the immune-inflammatory mechanism of DPN (Xue et al., 2021). Following oxidative stress, mitochondrial function is compromised, leading to an inadequate energy supply to neuronal axons, which consequently impairs nerve function (Eftekharpour and Fernyhough, 2022). The accumulation of inactive proteins due to protein peroxides in cells further impairs nerve function and diminishes neurotrophic support (Wang et al., 2005). Hyperglycemia triggers neuronal apoptosis through specific pathways (Liu et al., 2020). Research indicates that pressure on the skin can cause vasomotor dysfunction and inadequate blood flow adaptation, impeding normal capillary perfusion and resulting in ischemia and hypoxia of peripheral nerve tissues, ultimately leading to DPN (O'Brien, 2020). Some experts argue that the growth factors regulating neuronal growth play a role in the mechanism of DPN (Yan et al., 2018).

Second, according to Figure 7 and the keyword network map, “placebo-controlled trial” and “double-blind” are also key concepts, underscoring the clinical importance of double-blind, placebo-controlled trials in the context of DPN treatment. Although the concept of blind testing first emerged in the late 18th century, it took several decades to gain widespread acceptance. It was not until the 1940s and 1950s that Dr. Harry Gold, through extensive research publications, actively promoted and popularized the methodology of blind evaluation, firmly establishing it as a fundamental approach for validating treatment efficacy (Gold, 1954). A landmark double-blind, placebo-controlled trial for DPN was conducted by Rull et al. (1969), demonstrating the significant efficacy of carbamazepine compared to a placebo in treating DPN.

Finally, DPN is a chronic complication of diabetes mellitus. Currently, the clinical approach to treating DPN aligns with managing diabetes, emphasizing active and strict control of blood sugar levels, and addressing other underlying conditions through dietary regulation and moderate physical activity (Martin et al., 2014; Ishibashi et al., 2019). International guidelines (American Diabetes Association Professional Practice Committee, 2024) recommend a treatment strategy for diabetic patients that includes maintaining reasonable blood sugar levels, managing weight, and improving cardiovascular and renal outcomes. Therefore, effective blood sugar management is particularly crucial for patients with DPN. The following are the choices of drugs to control blood sugar: 1. Metformin (Inzucchi et al., 2014): by inhibiting hepatic glycogenolysis, enhancing glucose utilization in peripheral tissues, and promoting anaerobic glycolysis, among through other mechanisms, metformin effectively improves the prognosis of diabetes mellitus. Metformin is recommended as the first-line drug of choice in both domestic and international guidelines for type 2 diabetes mellitus (T2DM). It is also the preferred option for elderly diabetic patients (with no age restrictions) and can be used long-term (except in cases of renal insufficiency). 2. Alpha-glucosidase inhibitors (Kawamori et al., 2009): the *α*-glucosidase inhibitors effectively lower postprandial glucose levels by inhibiting the activity of intestinal glucosidases and delaying the absorption of carbohydrates from food. 3. Thiazolidinediones (TZDs) (Home et al., 2009): these medications reduce blood sugar levels by enhancing insulin sensitivity. 4.SGLT2i inhibitors (Gallo et al., 2015): by inhibiting the SGLT2 reabsorption of glucose in the proximal curved tubules of the kidney and increasing the excretion of glucose in the urine, blood sugar levels are lowered. 5. Incretin: ① GLP-1RA (Singh et al., 2017): these drugs exert a hypoglycemic effect by activating the GLP-1 receptor *in vivo*, enhancing insulin secretion, and inhibiting glucagon secretion in a glucose-dependent manner. ② DPP-4i (Mulvihill and Drucker, 2014): these medications improve glucose metabolism by increasing the body’s own GLP-1 level. 6. Insulin secretagogues: ① sulfonylureas (Müller et al., 2002): these drugs lower blood sugar levels by promoting the release of insulin from islet beta cells and ② non-sulfonylurea short-acting insulin secretagogues (Schwarz et al., 2008): these medications reduced PPG by stimulating early-phase insulin secretion. 7. Finally, insulin preparation: Insulin is the most effective hypoglycemic drug and is a life-saving necessity for people with severe hyperglycemia. However, side effects such as hypoglycemia and weight gain should be noted (Lee et al., 2012). In the treatment of DPN, in addition to glycemic control, other important approaches include etiologic treatment, neurotrophic repair, and symptomatic management. Over the past 4 years, “medication management” and “medication” have also been key areas of focus, with intensities of 5.82 and 5.36, respectively. Depending on the underlying cause, medications commonly prescribed to improve microcirculation include prostaglandin, beclomethasone sodium, cilostazol, hexeton theophylline, pancrealizine, and calcium dobesilate (Laczy et al., 2009). *α*-Lipoic acid is frequently used for its antioxidant properties (Hsieh et al., 2023), while the aldose reductase inhibitor, epalrestat, is used to treat metabolic disturbances (Li et al., 2016). For nerve repair and nutritional support, commonly used agents include methylcobalamin (Yao et al., 2016), nerve growth factor, gangliosides, inositol, and linolenic acid.

Furthermore, the keyword “duloxetine” shows a strength of 6.14 and is a tricyclic antidepressant (TCA). Based on a Class I meta-analysis, the EFNS has classified TCAs as Grade A evidence for their efficacy in treating DPN (Saarto and Wiffen, 2005). Other medications, such as pregabalin, gabapentin, lamotrigine, and sodium valproate, are also supported by Grade A evidence (Bragg et al., 2024). Furthermore, other studies indicate that opioids (Waldfogel et al., 2017) and serotonin–norepinephrine reuptake inhibitors (SNRIs) (Khdour, 2020) are also used in the treatment of DPN. Evidence-based guideline (Bril et al., 2011) also indicates that the above medications are currently the fundamental treatments for painful diabetic peripheral neuropathy.

In summary, compared to other bibliometric studies related to diabetes, this study specifically addressed various aspects of DPN treatment. The focus was clear, as it was dedicated to analyzing both past and present approaches to DPN treatment. The analysis indicated that, while pharmacotherapy remains the predominant approach, the literature shows that many other treatment methods are gradually being incorporated into clinical practice.

## 5 Future perspectives

Keywords and references with citation bursts can reflect the evolution and emerging trends in this scientific area (Ma et al., 2020; Fitzpatrick, 2005). Figure 9 shows the top 25 references with citation bursts. Among these, we have focused on the reference with highest strength, “Pharmacotherapy for neuropathic pain in adults: a systematic review and meta-analysis–2015” (strength 19.32), which is a position statement published in the *Lancet Neurol* in 2015 (Finnerup et al., 2015). It used the Grading of Recommendations Assessment, Development, and Evaluation (GRADE) framework to revise the Special Interest Group on Neuropathic Pain (NeuPSIG) recommendations for the pharmacotherapy of neuropathic pain, based on the results of a systematic review and meta-analysis. For the treatment of neuropathy, in addition to medications that help repair damaged nerves and promote nutritional nerve health, certain physical therapies have been found effective for DPN (Lepesis et al., 2023).

In additionresearch suggests that acupuncture can positively impact DPN alleviating various clinical symptoms inexpensively and with minimal side effects (Lin et al., 2021). It is important to consider that long-term use of medication may lead to dependency and potentially cause adverse reactions such as ataxia blurred vision constipation diplopia dizziness lethargy fatigue and nausea (Hor et al., 2018). When managing DPN it is crucial to prioritize strict control of blood sugar levels and targeted treatment of metabolic issues from the onset of diabetes diagnosis. Early implementation of neuroelectrophysiological examinations which are considered the gold standard for assessing DPN can effectively prevent and slow the progression of the condition in patients with DM (Iqbal et al., 2018). Prioritizing the detection of abnormalities and early intervention is essential for the effective management of DPN. It is recommended to use medications judiciously to minimize side effects in conjunction with complementary physical therapies for comprehensive care.

## 6 Strengths and limitations

To the best of our knowledge, this study is the first to review the research area of therapy and diabetic peripheral neuropathy from 2014 to 2024 through bibliometric analysis. The present study provides insights into growth trends and highlights current key challenges that require further investigation in this scientific area based on the published literature. This will enable researchers to better understand the latest developments and emerging topics in the field.

Nevertheless, this study has some limitations. First, the literature was searched exclusively in the database “Web of Science Core Collection” to ensure the quality of publications; however, this might have resulted in the exclusion of the other related publications, potentially leading to an incomplete analysis. Second, in the cluster analysis, we only provided information about nodes such as country, institution, author, and keyword, which might have resulted in the omission of other relevant information. In addition, the study excluded literature published after January 2024, which might have resulted in potential bias toward highly cited papers. Lastly, the search strategy might have been insufficiently comprehensive and the included literature might have differed significantly from the topic, leading to discrepancies between the retrieved results and the ideal outcomes.

## 7 Conclusion

This study provides an objective perspective on current treatments for DPN. The results indicate that drug therapy, “placebo-controlled trial,” and “oxidative stress” are current research hotspots. Meanwhile, the impact of “spinal cord stimulation” and “pain management” is gaining increasing attention from researchers and may emerge as future research trends. These findings provide valuable insights for further research and treatment of DPN.
